# Impact of Syndecan-2-Selected Mesenchymal Stromal Cells on the Early Onset of Diabetic Cardiomyopathy in Diabetic db/db Mice

**DOI:** 10.3389/fcvm.2021.632728

**Published:** 2021-05-21

**Authors:** Kathleen Pappritz, Fengquan Dong, Kapka Miteva, Arpad Kovacs, Muhammad El-Shafeey, Bahtiyar Kerim, Lisa O'Flynn, Stephen Joseph Elliman, Timothy O'Brien, Nazha Hamdani, Carsten Tschöpe, Sophie Van Linthout

**Affiliations:** ^1^Berlin Institute of Health at Charité – Universitätmedizin Berlin, BIH Center for Regenerative Therapies (BCRT), Berlin, Germany; ^2^Berlin-Brandenburg Center for Regenerative Therapies, Charité, Universitätsmedizin Berlin, Berlin, Germany; ^3^German Center for Cardiovascular Research (DZHK), Partner site Berlin, Berlin, Germany; ^4^Division of Cardiology, Foundation for Medical Research, Department of Medicine Specialized Medicine, Faculty of Medicine, University of Geneva, Geneva, Switzerland; ^5^Institute of Physiology, Ruhr University Bochum, Bochum, Germany; ^6^Medical Biotechnology Research Department, Genetic Engineering and Biotechnology Research Institute (GEBRI), City of Scientific Research and Technological Applications, Alexandria, Egypt; ^7^Orbsen Therapeutics, National University of Ireland Galway, Galway, Ireland; ^8^Regenerative Medicine Institute and Department of Medicine, National University of Ireland Galway, Galway, Ireland; ^9^Molecular and Experimental Cardiology, Ruhr University Bochum, Bochum, Germany; ^10^Department of Cardiology, St. Josef-Hospital, Ruhr University Bochum, Bochum, Germany; ^11^Department of Cardiology, Charité - Universitätsmedizin Berlin, Campus Virchow Klinikum, Berlin, Germany

**Keywords:** type 2 diabetes, diabetic cardiomyopathy, syndecan-2/CD362^+^-selected stromal cells, immunomodulation, cardiomyocyte stiffness, cardiac fibrosis, angiogenesis

## Abstract

**Background:** Mesenchymal stromal cells (MSCs) are an attractive cell type for cell therapy given their immunomodulatory, anti-fibrotic, and endothelial-protective features. The heparin sulfate proteoglycan, syndecan-2/CD362, has been identified as a functional marker for MSC isolation, allowing one to obtain a homogeneous cell product that meets regulatory requirements for clinical use. We previously assessed the impact of wild-type (WT), CD362^−^, and CD362^+^ MSCs on local changes in protein distribution in left ventricular (LV) tissue and on LV function in an experimental model of early-onset diabetic cardiomyopathy. The present study aimed to further explore their impact on mechanisms underlying diastolic dysfunction in this model.

**Materials:** For this purpose, 1 × 10^6^ WT, CD362^−^, or CD362^+^ MSCs were intravenously (i.v.) injected into 20-week-old diabetic BKS.Cg-m+/+Lepr^db^/BomTac, i.e., db/db mice. Control animals (db+/db) were injected with the equivalent volume of phosphate-buffered saline (PBS) alone. After 4 weeks, mice were sacrificed for further analysis.

**Results:** Treatment with all three MSC populations had no impact on blood glucose levels in db/db mice. WT, CD362^−^, and CD362^+^ MSC application restored LV nitric oxide (NO) and cyclic guanosine monophosphate (cGMP) levels in db/db mice, which correlated with a reduction in cardiomyocyte stiffness. Furthermore, all stromal cells were able to increase arteriole density in db/db mice. The effect of CD362^+^ MSCs on NO and cGMP levels, cardiomyocyte stiffness, and arteriole density was less pronounced than in mice treated with WT or CD362^−^ MSCs. Analysis of collagen I and III protein expression revealed that fibrosis had not yet developed at this stage of experimental diabetic cardiomyopathy. All MSCs reduced the number of cardiac CD3^+^ and CD68^+^ cells in db/db mice, whereas only splenocytes from CD362^−^- and CD362^+^-db/db mice exhibited a lower pro-fibrotic potential compared to splenocytes from db/db mice.

**Conclusion:** CD362^+^ MSC application decreased cardiomyocyte stiffness, increased myocardial NO and cGMP levels, and increased arteriole density, although to a lesser extent than WT and CD362^−^ MSCs in an experimental model of early-onset diabetic cardiomyopathy without cardiac fibrosis. These findings suggest that the degree in improvement of cardiomyocyte stiffness following CD362^+^ MSC application was insufficient to improve diastolic function.

## Introduction

Diabetes mellitus is a global health problem, and despite enormous advances in therapy options and patient self-management, over 600 million people worldwide are expected to be affected by 2045 (1). Diabetic cardiomyopathy is an own clinical entity, which is characterized by structural and functional alterations of the heart (2), including interstitial inflammation (3), cardiac fibrosis (4), impaired cardiac vascularization (5), and cardiomyocyte stiffness (6). Due to excessive collagen deposition (7, 8) and cardiomyocyte stiffness (9, 10), left ventricular (LV) function is impaired, although cardiomyocyte stiffness alone is sufficient to induce LV diastolic dysfunction without any involvement of collagen (10, 11). Alterations in the NO–cyclic guanosine monophosphate (cGMP)–protein kinase (PK)G-titin phosphorylation pathway have been identified as underlying mechanisms of abnormal cardiomyocyte stiffness in diabetic mice (11, 12), rats (13, 14), and patients suffering from heart failure with preserved ejection fraction (13–15).

Bone marrow-derived mesenchymal stromal cells (MSCs) are an attractive tool to treat diabetic cardiomyopathy due to their immunomodulatory properties (16, 17), their capacity to home to damaged tissues (17), their low immunogenic nature (18), and their anti-diabetic properties (19, 20). With respect to cardiac repair, MSCs have been demonstrated to differentiate into cardiomyocytes, endothelial cells, and smooth muscle cells (21), but their cardioprotective effects, including immunomodulatory (22–24), anti-fibrotic (17), and pro-angiogenic effects (11) have mainly been attributed to their paracrine actions. This also includes the ability of MSCs to restore impaired titin phosphorylation and hereto-related cardiomyocyte stiffness and diastolic dysfunction (11, 25).

A potential concern in the use of MSCs as a therapeutic cellular product is the isolation technique, which relies on the plastic adherence of bone marrow mononuclear cells (MNCs) and leads to a heterogeneous population. Although minimal criteria for MSC characterization have been defined (26), this still heterogeneous human MSC population may not be sufficiently pure to meet emerging regulatory requirements for Advanced Therapeutic Medicinal Products for clinical use. Syndecan-2/CD362, a heparin sulfate proteoglycan, is expressed on the surface of a subpopulation of human MSCs and allows the selective isolation of these cells via fluorescence-activated cell sorting.

In experimental *Escherichia coli*-induced pneumonia, Masterson et al. (27) demonstrated that CD362^+^-selected MSCs decreased pneumonia severity and that their efficacy was at least comparable with that of heterogeneous MSCs. We recently assessed local changes in protein distribution in myocardial tissue in response to wild-type (WT), CD362^+^, and CD362^−^-selected cell application in db/db mice (25). Subsequent investigations of the phosphorylation state of titin in the heart revealed that only WT and CD362^−^-selected MSCs restored all-titin phosphorylation and downstream PKG activity in db/db mice, which were paralleled with an improvement in parameters of diastolic function.

With CD362^+^-selected MSCs being a homogeneous cell product that meets regulatory requirements for clinical use, the present study aimed to gain further insights into the impact of CD362^+^-selected MSC application on the pathogenesis of experimental diabetic cardiomyopathy, especially on the mechanisms underlying diastolic dysfunction. To this end, CD362^+^, CD362^−^, and WT MSCs were intravenously (i.v.) administrated in type 2 diabetic db/db mice and their impact on cardiomyocyte stiffness, myocardial NO and cGMP levels, cardiac fibrosis, cardiac immune cell presence, and angiogenesis was analyzed.

## Materials and Methods

### Mesenchymal Stromal Cell Preparation

As described in detail previously (25, 27), bone marrow-derived WT MSCs (WT), CD362^−^ MSCs (CD362^−^), and CD362^+^ MSCs (CD362^+^) were obtained from Orbsen Therapeutics Ltd. (Galway, Ireland). Via Ficoll density gradient centrifugation (GE Health Care Bio-Sciences, Buckinghamshire, UK), MNCs were isolated, followed by lysis of erythrocytes using commercially available ACK lysis buffer (Life Technologies, California, USA). Subsequently, cells were stained with antibodies directed against CD23 (eBioscience, Hatfield, UK), CD45 (BD Biosciences, Oxford, UK), CD271 (Miltenyi Biotec, Bergisch Gladbach, Germany), and CD362 (R&D Systems, Abingdon, UK). As a marker for cell viability, Sytox Blue (Life Technologies, California, USA) was used. On a BD FACS Aria (BD Biosciences, Oxford, UK), cells were sorted and plated afterwards for further expansion. At passage 2, the different cell types were cryopreserved and shipped to the consortia partners for further experiments. Informed consent was obtained for all bone marrow samples (Ethics Ref. C.A.02/08).

### Model of Experimental Type 2 Diabetes Mellitus-Associated Diabetic Cardiomyopathy

As a common model for human type 2 diabetes mellitus, leptin receptor (Lepr) knockout mice, commonly known as db/db mice, were used. In detail, 8-week-old, male heterozygous (db+/db) and homozygous (db/db) BKS.Cg-m+/+Lepr^db^/BomTac mice were purchased from Taconic (Skensved, Denmark) and housed in the animal facilities of the Charité-Universitätsmedizin Berlin under standard housing conditions (12-h light/dark cycle, 50–70% humidity, 19–21°C) with free access to food and water. At the age of 20 weeks, db+/db and db/db mice were randomly divided over the groups and administered with 1 × 10^6^ WT, CD362^−^, or CD362^+^ MSCs in 200 μl of phosphate-buffered saline (PBS) via i.v. injection. Control animals received the same volume (200 μl) of PBS alone (Life Technologies, Darmstadt, Germany). The respective n-number of each group is indicated in the figure legends. Four weeks after cell or PBS application, at the age of 24 weeks, mice were anesthetized with a mixture of buprenorphine [0.05 mg/kg bodyweight (BW)] and urethane [0.8–1.2 g/kg BW] to enable subsequent characterization of LV function (25). After functional characterization, mice were sacrificed via cervical dislocation under anesthesia. Subsequently, organs of interest were harvested for further analysis. All experimental procedures were performed according to the European legislation (Directive 2010/63/EU) and approved by the local animal welfare committee (Landesamt für Gesundheit und Soziales, Berlin, Germany, G0254/13).

### Blood Glucose and Determination of Glycated Hemoglobin

Before and once a week after cell application, blood glucose (BG) was measured using the Accu-Chek Aviva^®^ (Roche Diabetes Care Deutschland GmbH, Mannheim, Germany) after 4-h fasting. To quantify the glycated hemoglobin fraction HbA_1c_ in whole blood samples, the Helena GLYCO-Tek kit (Helena Laboratories, Texas, USA) was used. In accordance with the manufacturer's protocol, blood samples were hemolyzed with the hemolysate reagent and subsequently vortexed. Next, samples were loaded on special GLYCO-Tek affinity columns placed on collection tubes. Columns were washed with Developer A, and non-glycated hemoglobin (GHb) was eluted by adding 4 ml of Developer A afterwards. The resulting eluate was adjusted to 15 ml with deionized water containing non-GHb. Subsequently, Developer B was used to extract GHb from the same sample. After three times' inversion, the solution of the collection tubes was transferred to a cuvette and measured photometrically at 415 nm on a Spectra Max 340PC microplate reader (Molecular Devices, Biberach an der Riß, Germany). The calculated percentage (%) of GHb was transferred into the percentage (%) of HbA_1c_ using the provided algorithm.

### Tissue Preparation

After hemodynamic measurement (25), blood was taken and stored on ice until centrifugation. Additionally, the respective tissues were harvested and immediately snap frozen in liquid nitrogen. For flow cytometry, spleens were collected and stored on ice until further processing.

### Assessment of Human Mesenchymal Stromal Cells After Application in Different Organs

In accordance to McBride et al. (28), the enrichment of the human MSCs in different organs was investigated by Alu-PCR. Genomic DNA from frozen LV, lung, kidney, liver, spleen, and pancreas was extracted. As previously described (11, 29), human genomic DNA obtained from human umbilical vein endothelial cells served as standard, which was serially diluted over a 100,000-fold range, into murine spleen genomic DNA. To perform real-time PCR, 800 ng of target DNA, Alu-specific primers, and a fluorescent probe were used. Consistent with findings of Lee et al. (30), human DNA was not detectable 4 weeks after application in any of the analyzed organs/tissues (data not shown).

### Passive Force Measurements of Isolated Cardiomyocytes

To record passive force (F_passive_) of skinned cardiomyocyte preparations, frozen LV samples were first mechanically disrupted in relaxing solution, containing 1.0 mM of Mg^2+^, 100 mM of KCl, 2.0 mM of EGTA, 4.0 mM of Mg-ATP, and 10 mM of imidazole (all Sigma-Aldrich, Munich, Germany), followed by incubation in relaxing solution supplemented with 0.5% Triton X-100 for 5 min. Next, five times' washing in relaxing solution was performed. Under an inverted microscope (Zeiss Axiovert 135, 40 × objective; Carl Zeiss AG Corp., Oberkochen, Germany), single cardiomyocytes were selected and subsequently fixed in a “Permeabilized Myocyte Test System” (1600A; with force transducer 403A; Aurora Scientific, Aurora, ON, Canada) using silicone adhesive. F_passive_ was measured in relaxing buffer by stepwise stretching of the cardiomyocytes within a sarcomere length (SL) range between 1.8 and 2.4 μm. All measurements were performed at room temperature. For data presentation and analysis, force values were normalized to myocyte cross-sectional area (12).

### Determination of Myocardial Nitric Oxide Levels

To determine the concentration of myocardial NO, a colorimetric assay kit (BioVision Inc., Milpitas, CA, USA) was used. As described previously (12), frozen LV samples were first treated with trichloroacetic acid (Sigma-Aldrich), washed with 1 ml of 0.2% dithiothreitol, and subsequently homogenized in 1% sodium dodecyl sulfate (SDS) sample buffer, containing tri-distilled water, glycerol, SDS, Tris-HCl, brome-phenol blue, and DTT (all purchased from Sigma-Aldrich). After centrifugation at 14,000*g* for 15 min (2–8°C), absorbance of the supernatants was measured at 570 nm. As standard curve, an assay buffer was used from which the absorbance of the samples could be translated into nitrate/nitrite concentrations (15).

### Determination of Myocardial Cyclic Guanosine Monophosphate Concentration

Cardiac cGMP levels were investigated by the use of the commercially available parameter cGMP assay immunoassay kit (R&D systems, Minneapolis, MN, USA). In brief, homogenates of frozen LV samples were prepared and subsequently diluted in cell lysis buffer to a concentration of 0.025 μg/L. In accordance with the manufacturer's protocol, 100 μl of the homogenate was assayed by which the amount of cGMP present in the homogenate competed with fixed amount of horseradish peroxidase-labeled cGMP for sites on a rabbit polyclonal antibody. All samples were analyzed in duplicate, and cGMP concentration was expressed in pmol/ml (14).

### Immunohistochemistry

Frozen LV samples were embedded in Tissue-Tek OCT (Sakura, Zoeterwoude, NL) and cut into 5-μm-thick sections. Subsequently, immunohistological stainings using the avidin–biotin complex (ABC) method or the EnVision^®^ method were performed. To this end, the following antibodies were used: anti-collagen I (dilution 1:350; Chemicon, Limburg, Germany), anti-collagen III (dilution 1:200; Calbiochem, Merck Millipore, Darmstadt, Germany), anti-CD3 (dilution 1:35; Santa Cruz Biotechnology, Heidelberg, Germany), anti-CD4 (dilution 1:50; BD Bioscience/Pharmigen, Heidelberg, Germany), anti-CD8a (dilution 1:50; BioLegend, Koblenz, Germany), and anti-CD68 (dilution 1:600; Abcam, Cambridge, Germany). The EnVision^®^ method was used to investigate cardiac collagen I, collagen III, and α-SMA expression. To determine the presence of inflammatory cells such as CD3, CD4, CD8a, and CD68 in LV tissue samples, the ABC method was performed. In general, specific epitopes of the stained structure are colored red, and the heart area (HA) was counterstained with Heamalaun (blue). Quantitative analysis of all stainings was performed at 200× magnification in a blinded manner by digital image analysis on a Leica DM2000 light microscope (Leica Microsystems, Wetzlar, Germany). For this purpose, special macros were created to assess the number of cells/HA (mm^2^) or the positive area (%)/HA (mm^2^). In total, a minimum of 50 pictures from two LV sections from different cutting levels for each animal were recorded and analyzed. To assess artery and arteriole density, an anti-α-smooth muscle actin (α-SMA) antibody (dilution 1:200; Abcam) was used. In 10 high-power field (hpf), arteries and arterioles were counted at 100× magnification and presented as number of arteries or arterioles per hpf.

### Flow Cytometry

Splenocytes were isolated according to Van Linthout et al. (22). In brief, collected spleens of PBS- and cell-treated mice were positioned in a petri dish containing Roswell Park Memorial Institute (RPMI) media (Life Technologies). Next, spleens were mashed through a 70-μm cell strainer, followed by washing with 1% fetal bovine serum (FBS; Biochrom, Berlin, Germany) in PBS (Life Technologies). After centrifugation, supernatant was carefully aspirated, and the remaining pellet was resuspended in ACK lysis buffer (Life Technologies). Erythrocyte lysis was stopped by adding RPMI (Life Technologies), and the cell suspension was subsequently passed through a 40-μm cell strainer. Next, the cell suspension was again centrifuged, and the remaining cell pellet was resuspended for subsequent staining.

To assess the number of splenic (apoptotic; Annexin V^+^) regulatory T cells (Tregs; CD4^+^CD25^+^FoxP3^+^ cells), splenocytes were incubated with anti-CD4 FITC (dilution 1:20; Miltenyi Biotec), anti-CD25 PE (dilution 1:20; Miltenyi Biotec), and anti-Annexin V V450 (dilution 1:20; BD Bioscience, Heidelberg, Germany) antibodies for 20 min at 4°C in the dark. Subsequently, cells were washed and resuspended in fixation/permeabilization solution (BD Biosciences, New Jersey, USA). Finally, incubation with an anti-FoxP3 APC (dilution 1:10; Miltenyi Biotec) antibody followed. To determine the percentage of IL-10 and IFN-γ expressing CD4^+^ and CD8^+^ cells, splenocytes were stimulated after isolation with Iscove medium (Sigma-Aldrich) containing 50 ng/ml of phorbol 12-myristate 13-acetate (PMA; BD Biosciences, Heidelberg, Germany), 500 ng/ml of Ionomycin (BD Biosciences), and GolgiStop™ (dilution 1:1,500; BD Biosciences) prior to staining with anti-CD4 FITC and anti-CD8 VioBlue (both dilution 1:10; both Miltenyi). Afterwards, cells were fixated and permeabilized before they were incubated with the respective anti-IFN-γ APC and IL-10 PE (both dilution 1:10; both Miltenyi) antibodies.

After the respective staining was finished, cells were resuspended in PBS and measured on a MACSQuant Analyzer (Miltenyi Biotec). For data analysis, the FlowJo software version 8.8.6 (Tree Star Inc.) was used.

### RNA Isolation From Left Ventricular Tissue and Gene Expression Analysis

As previously published (23), RNA was isolated using the TRIzol™ method (Invitrogen, Heidelberg, Germany). To this end, frozen LV tissue was homogenized in TRIzol™ reagent, followed by chloroform extraction. Next, RNA was precipitated and subsequently purified using the NucleoSpin^®^ RNA mini kit (Macherey-Nagel GmbH, Düren, Germany) according to the manufacturer's protocol. The concentration of RNA was measured at the absorbance of 260 nm by using a NanoDrop 1000 (Thermo Scientific, Erlangen, Germany). For subsequent cDNA transcription, 1 μg of RNA and the High Capacity cDNA Reverse Transcription Kit from Applied Biosystems (Life Technologies GmbH) was used. Expression levels of the respective target genes were examined on a 7900HT real-time system (Applied Biosystems). Therefore, gene reporter assays from Applied Biosystems for lysyl oxidase (Lox; Mm00495386_m1), lysyl oxidase like (Loxl)-2 (Mm00804740_m1), transforming growth factor-β (TGF-β; Mm00441724_m1), and GAPDH (Mm99999915_g1) as a housekeeping gene were used. To examine the n-fold change, mRNA levels were further normalized to the db+/db group set as 1.

### Co-culture of Fibroblasts With Splenocytes

To determine the impact of splenocytes on collagen production in fibroblasts, splenocytes of the different experimental groups were co-cultured with murine C4 fibroblasts. According to Van Linthout et al. (11), fibroblasts were plated at a density of 10,000 cells per well in Iscove Basal Medium (Sigma-Aldrich) containing 10% FBS and 1% penicillin/streptomycin (both Biochrom). Twenty-four hours later, isolated splenocytes were added at a ratio of 1 to 10 (fibroblasts to splenocytes) in Iscove medium (Sigma) supplemented with 10% FBS and 1% penicillin/streptomycin (both Biochrom) in the presence of 50 ng/ml of PMA and 500 ng/ml of Ionomycin (both BD Biosciences). After 24-h co-culture, splenocytes were removed, and fibroblasts were fixed with cold methanol. Subsequently, Sirius red staining was performed. For photometric analyses at 540 nm, the Spectra Max 340PC microplate reader (Molecular Device GmbH) was used.

### Statistical Analysis

All data are illustrated as mean ± SEM, and statistical analysis was performed using the GraphPad Prism 9 Software (GraphPad Software, La Jolla, USA). Differences were considered significant at *p* < 0.05 using one-way ANOVA with Fisher's least significant difference (LSD) *post hoc* test (parametric data) or Brown–Forsythe and Welch-ANOVA followed by unpaired *t*-test with Welch's correction (non-parametric data).

## Results

### Application of Wild-Type, CD362^−^, and CD362^+^ Mesenchymal Stromal Cells Does Not Affect Blood Glucose and HbA_1c_ Levels of db/db Mice

BG (Supplementary Figures 1A,B) and HbA_1c_ levels (Supplementary Figure 1C) were elevated in db/db mice compared with db+/db mice. None of the applied MSCs reduced BG or HbA_1c_ levels in db/db mice (Supplementary Figures 1B,C).

### Application of Wild-Type, CD362^−^, and CD362^+^ Mesenchymal Stromal Cell Reduces Cardiomyocyte Stiffness in db/db Mice

**Figure 1:**
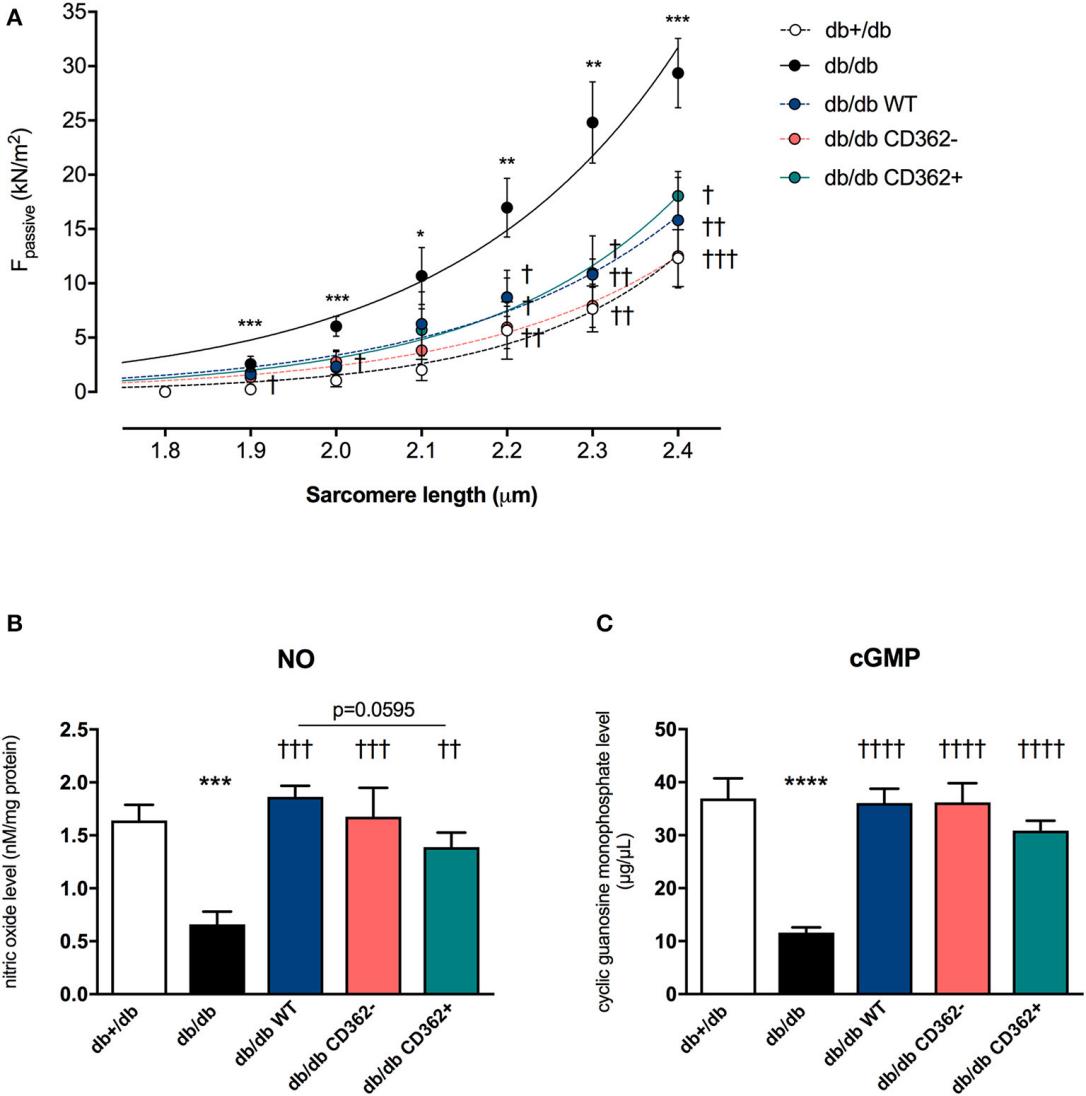
Application of wild-type (WT), CD362^−^, and CD362^+^ mesenchymal stromal cells (MSCs) reduces cardiomyocyte stiffness in db/db mice. **(A)** Passive force (F_passive_; in kN/m^2^) measurements in single cardiomyocytes at different sarcomere length (1.8–2.4 μm). To further investigate the underlying NO-cGMP-PKG pathway, cardiac nitric oxide (NO; **B**; in nM/mg protein) and cyclic guanosine monophosphate (cGMP; **C**; in μg/μl) concentration in left ventricular (LV) tissue homogenates were measured. Bar graphs represent the mean ± SEM and were analyzed with one-way ANOVA or Welch-ANOVA (*p < 0.05, **p < 0.01, ***p < 0.001, ****p < 0.0001 vs. db+/db; ^†^*p* < 0.05, ^††^*p* < 0.01, ^†††^*p* < 0.001, ^††††^*p* < 0.0001 vs. db/db; with *n* = 8/group for **A**; *n* = 5/group for **B,C**).

Previous studies have shown that application of WT and CD362^−^ MSCs improves diastolic function in db/db mice, whereas application of CD362^+^ MSCs does not (25). To gain further insights into the mechanisms underlying their impact on diastolic dysfunction, *ex vivo* measurements of cardiomyocyte stiffness were performed (Figure 1A). In accordance with the literature (12), db/db mice showed increased F_passive_ in isolated cardiomyocytes compared with control animals. Interestingly, all cell types reduced F_passive_. This reduction was the most pronounced in the db/db CD362^−^ group and less prominent in the db/db CD362^+^ group. Since regulation of titin phosphorylation via NO-cGMP-PKG signaling (12) is important for proper diastolic function (14), we therefore measured the levels of myocardial NO and cGMP (Figures 1B,C). db/db mice displayed reduced NO and cGMP levels than did db+/db animals, which correlates with the higher F_passive_ values observed in db/db mice. Intravenous administration of WT, CD362^−^, and CD362^+^ MSCs restored the reduction of NO and cGMP levels in db/db mice (Figures 1B,C), by which this effect was less pronounced in db/db mice receiving CD362^+^ MSCs.

### Application of Wild-Type, CD362^−^, and CD362^+^ Mesenchymal Stromal Cell Does Not Influence Cardiac Collagen Expression in db/db Mice

**Figure 2:**
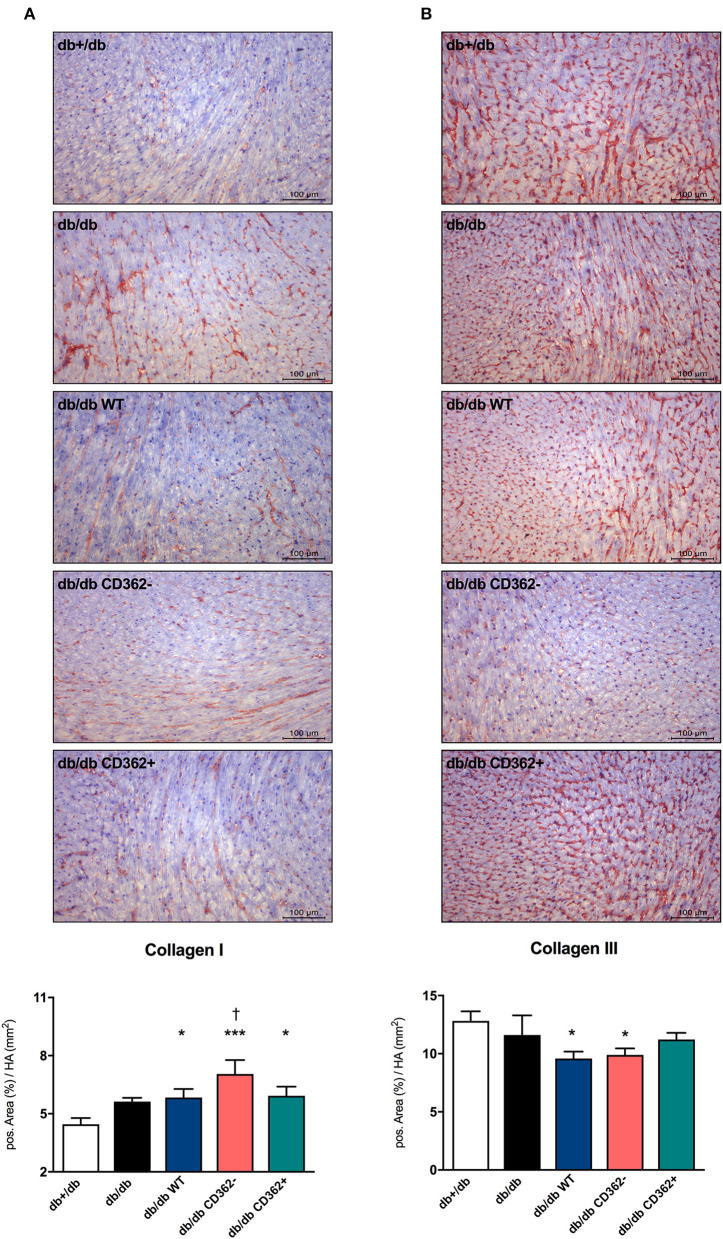
Application of wild-type (WT), CD362^−^, and CD362^+^ mesenchymal stromal cells (MSCs) does not influence cardiac collagen expression in db/db mice. Representative images (scale bar = 100 μm) of the cardiac collagen I **(A)** and collagen III **(B)** staining and the corresponding quantification of the positive area (%)/heart area (HA; mm^2^). Data are depicted as mean ± SEM and analyzed with one-way ANOVA or Welch-ANOVA (*p < 0.05, ***p < 0.001, vs. db+/db; ^†^*p* < 0.05 vs. db/db; with *n* = 11 for db+/db, *n* = 9 for db/db, *n* = 9 for db/db WT, *n* = 9 for db/db CD362^−^, and *n* = 10 for db/db CD362^+^).

Given the importance of cardiac fibrosis in diabetic cardiomyopathy (3) and diastolic dysfunction (7) and the anti-fibrotic potential of MSCs (17), the impact of MSC application on cardiac fibrosis was analyzed (Figure 2 and Supplementary Figure 2). Quantitative analysis of collagen I and III revealed no significant changes in collagen I and III protein expression between db/db and db+/db control mice (Figures 2A,B), indicative of no developed cardiac fibrosis at this stage in db/db mice. However, the collagen I/III protein ratio was increased in db/db animals vs. db+/db mice, showing alterations in the extracellular matrix composition (Supplementary Figure 2A). Application of WT, CD362^−^, and CD362^+^ MSCs resulted in a higher LV collagen I protein expression compared with db+/db mice, whereas WT and CD362^−^ MSCs reduced LV collagen III protein expression than did db+/db controls (Figures 2A,B). The collagen I/III protein ratio was increased in db/db mice, which received WT, CD362^−^, and CD362^+^ MSCs as compared with db+/db mice (Supplementary Figure 2A). In addition to cardiac collagen content, the gene expression of known fibrosis mediator TGF-β (Supplementary Figure 2B) and the collagen crosslinking enzymes Lox (Supplementary Figure 2C) and LoxL-2 (Supplementary Figure 2D) were also determined. In comparison with db+/db mice, TGF-β, Lox, and LoxL-2 mRNA levels were elevated in db/db animals. None of the different stromal cells reduced TGF-β, Lox, and LoxL-2 gene expression in db/db mice.

### Application of Wild-Type, CD362^−^, and CD362^+^ Mesenchymal Stromal Cell Increases Arteriole Density in db/db Mice

**Figure 3:**
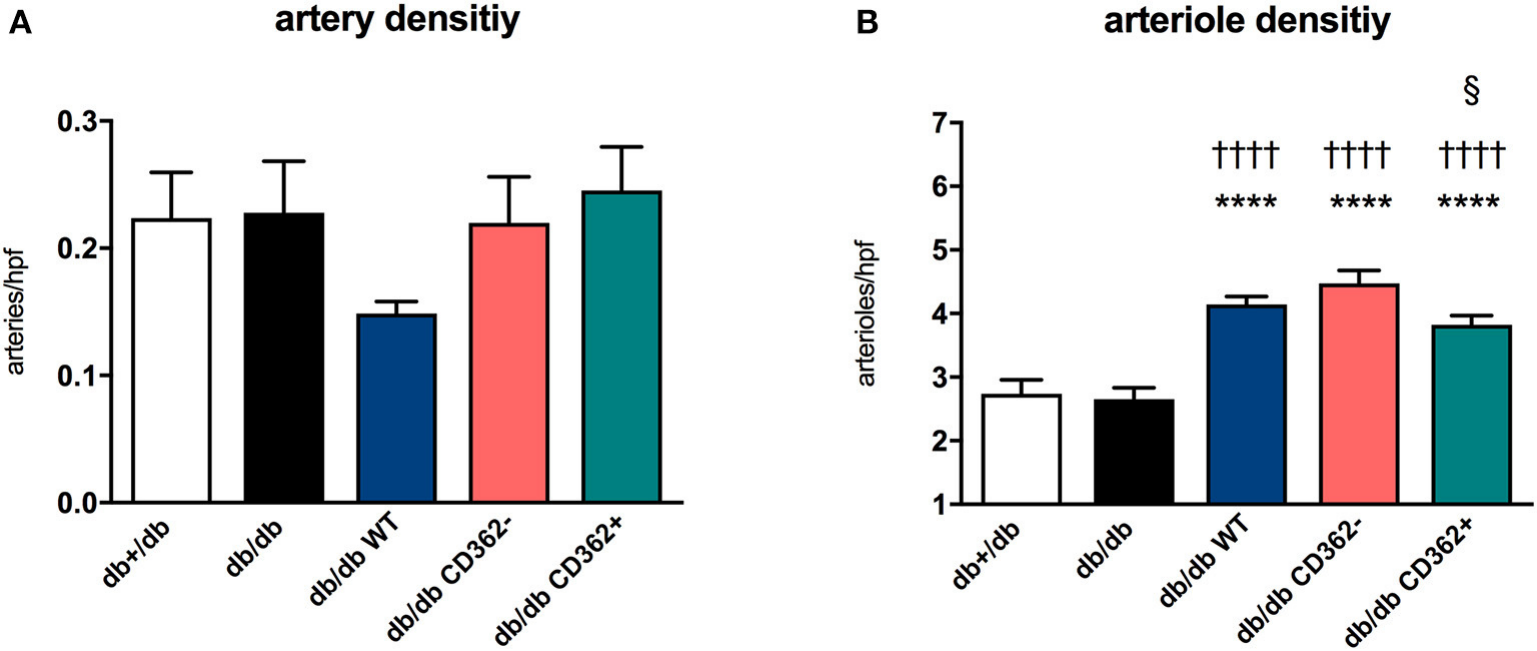
Application of wild-type (WT), CD362^−^, and CD362^+^ mesenchymal stromal cells (MSCs) increases arteriole density in db/db mice. **(A)** Artery and **(B)** arteriole density per high-power field (hpf) in db+/db, db/db, and db/db mice injected with WT, CD362^−^, or CD362^+^ cells. Bar graphs represent the mean ± SEM and were analyzed with one-way ANOVA or Welch-ANOVA (****p < 0.0001 vs. db+/db; ^††††^*p* < 0.0001 vs. db/db; ^§^*p* < 0.05 vs. db/db CD362^−^ with *n* = 8 for db+/db, *n* = 9 for db/db, *n* = 8–9 for db/db WT, *n* = 9–10 for db/db CD362^−^, and *n* = 10–11 for db/db CD362^+^ group).

Since impaired vascularization underlies diastolic dysfunction (8, 31) and MSCs are known for their pro-angiogenic properties (11), we next evaluated the impact of WT, CD362^−^, and CD362^+^ MSC application on artery and arteriole density in LV sections of db/db mice (Figure 3). There was no decrease in artery and arteriole density in db/db mice compared with db+/db mice (Figures 3A,B). Administration of WT, CD362^−^, and CD362^+^ MSCs resulted in a higher arteriole density in db/db mice and was the most pronounced in db/db WT and db/db CD362^−^-treated mice (Figure 3B).

### Application of Wild-Type, CD362^−^, and CD362^+^ Mesenchymal Stromal Cell Influences Cardiac Immune Cell Presence in db/db Mice

**Figure 4:**
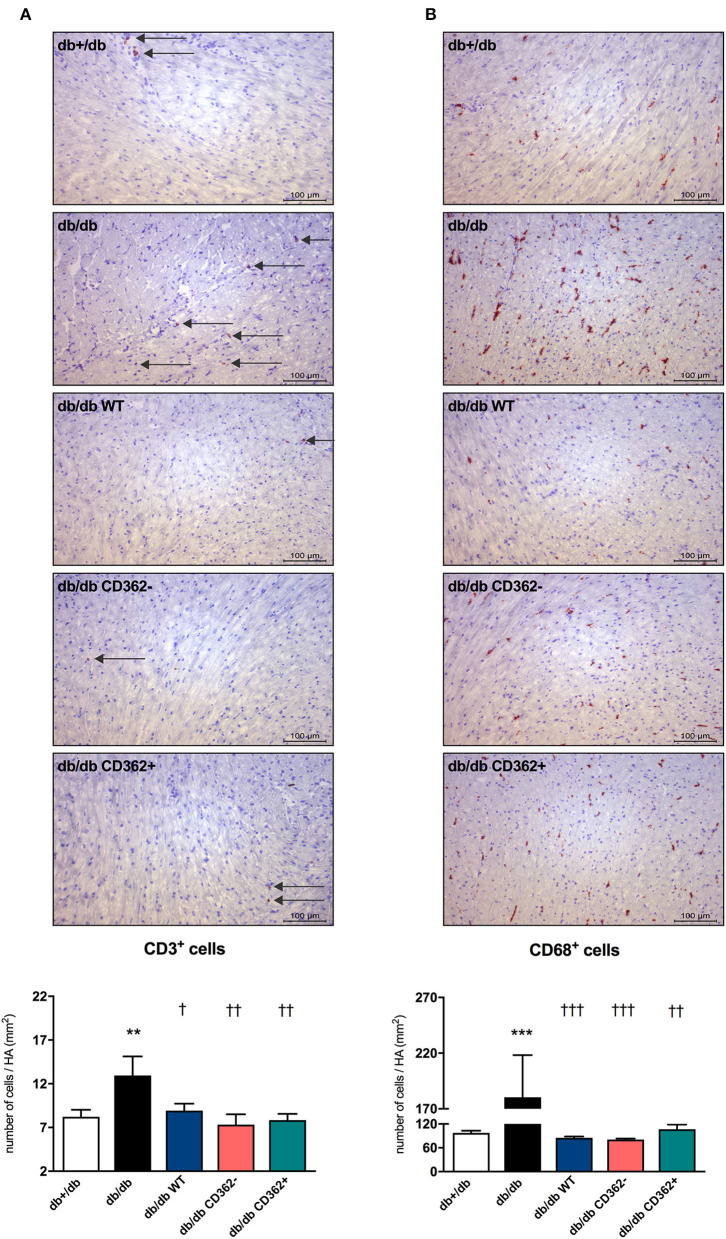
Application of wild-type (WT), CD362^−^, and CD362^+^ mesenchymal stromal cells (MSCs) influences cardiac presence of CD3^+^ and CD68^+^ immune cells in db/db mice. Characterization of the immune cells present within the myocardium 4 weeks after stromal cell administration. Top: representative immunohistological stainings (scale bar = 100 μm) of CD3^+^ **(A)** and CD68^+^ **(B)** cells. The specific epitopes are colored red and indicated by the arrows. Bottom: the respective quantification performed via digital image analysis is depicted. Bar graphs represent the mean ± SEM of the number of cells/heart area (HA; mm^2^) and were analyzed with one-way ANOVA or Welch-ANOVA (**p < 0.01, ***p < 0.001 vs. db+/db; ^†^*p* < 0.05, ^††^*p* < 0.01, ^†††^*p* < 0.001 vs. db/db with *n* = 10–11 for db+/db, *n* = 6–7 for db/db, *n* = 7–9 for db/db WT, *n* = 7–8 for db/db CD362^−^, and *n* = 11 for db/db CD362^+^ group).

Given the importance of cardiac inflammation in diabetic cardiomyopathy (32) and the immunomodulatory properties of MSCs (33), we next evaluated the impact of i.v. WT, CD362^−^, and CD362^+^ MSC application on cardiac immune cell presence. Therefore, the number of CD3^+^, CD4^+^, CD8a^+^, and CD68^+^ cells was assessed in the myocardium of the different groups (Figure 4 and Supplementary Figure 3). Compared with db+/db mice, db/db animals displayed an increased number of CD3^+^ (Figure 4A), CD4^+^ (Supplementary Figure 3A), CD8a^+^ (Supplementary Figure 3B), and CD68^+^ (Figure 4B) cells in the heart. Administration of WT, CD362^−^, and CD362^+^ MSCs abrogated the number of cardiac CD3^+^ and CD68^+^ cells in db/db animals to levels similar to those of non-diabetic db+/db mice (Figures 4A,B), whereas none of the stromal cells resulted in lower CD4^+^ and CD8a^+^ cells in db/db mice (Supplementary Figures 3A,B).

### Application of Wild-Type, CD362^−^, and CD362^+^ Mesenchymal Stromal Cell Modulates Splenic Immune Cells in db/db Mice

**Figure 5:**
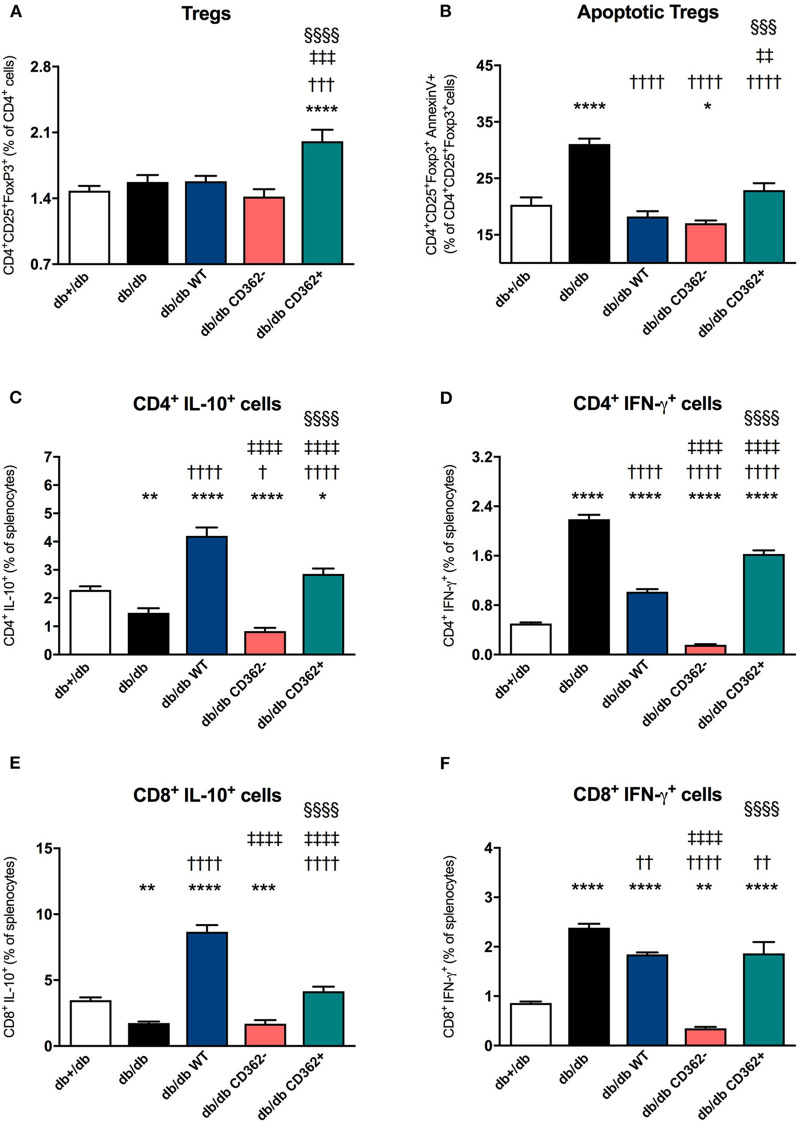
Application of wild-type (WT), CD362^−^, and CD362^+^ mesenchymal stromal cells (MSCs) modulates the composition of splenic immune cells in db/db mice. To investigate if stromal cell application influences splenic immunomodulation, flow cytometry was performed; and the % of regulatory T cells (Tregs; **A**) and apoptotic Tregs **(B)** was determined. Additionally, anti-inflammatory CD4^+^IL-10^+^ **(C)** and CD8^+^IL-10^+^ **(D)** cells and their pro-inflammatory counterparts, CD4^+^IFN-γ^+^ **(E)**, and CD8^+^IFN-γ^+^ **(F)** cells were analyzed by flow cytometry. Data are depicted as mean ± SEM and analyzed with one-way ANOVA or Welch-ANOVA (*p < 0.05, **p < 0.01, ***p < 0.001, ****p < 0.0001 vs. db+/db; ^†^*p* < 0.05, ^††^*p* < 0.01, ^†††^*p* < 0.001, ^††††^*p* < 0.0001 vs. db/db; ^‡‡^*p* < 0.01, ^‡‡‡^*p* < 0.001, ^‡‡‡‡^*p* < 0.0001 vs. db/db WT; ^§§§^*p* < 0.001, ^§§§§^*p* < 0.0001 vs. db/db CD362^−^ with *n* = 5–6/group).

Based on the knowledge that the cardiosplenic axis, which describes the homing of immune cells from the spleen toward the heart, has an impact on the progression of heart failure (34), we further evaluated whether WT, CD362^−^, and CD362^+^ MSC application affects splenic immune cells in db/db mice (Figure 5). By an unchanged percentage of splenic CD4^+^CD25^+^FoxP3^+^ cells (Tregs) (Figure 5A), db/db mice exhibited a higher percentage of splenic apoptotic Tregs vs. db+/db mice (Figure 5B). Furthermore, the amount of anti-inflammatory CD4^+^IL-10^+^ and CD8^+^IL-10^+^ cells was lower in the spleen of db/db mice vs. db+/db animals (Figures 5C,E), which was paralleled by an increased amount of splenic pro-inflammatory CD4^+^IFN-γ^+^ and CD8^+^IFN-γ^+^ cells (Figures 5D,F). Application of WT, CD362^−^, and CD362^+^ MSC cell in db/db mice resulted in a lower percentage of apoptotic Tregs compared with db/db mice (Figure 5B). WT and CD362^+^ cells resulted in a higher number of splenic CD4^+^IL-10^+^ and CD8^+^IL-10^+^ cells in db/db mice, whereas CD362^−^ cells did not alter those cell populations (Figures 5C,E). In contrast, a decrease in CD4^+^IFN-γ^+^ and CD8^+^IFN-γ^+^ cells was the most pronounced after CD362^−^ MSC administration (Figures 5D,F).

Knowing that splenocytes play an important role in cardiac remodeling (34) and the above-described systemic immunomodulation, we finally evaluated the impact of MSC application on the pro-fibrotic potential of splenocytes (Supplementary Figure 4). Therefore, splenocytes of the different groups were co-cultured with fibroblasts, and their subsequent impact on collagen production was determined (Supplementary Figure 4A). Upon co-culture, collagen production was increased in fibroblasts supplemented with splenocytes from db/db mice compared with fibroblasts supplemented with splenocytes from db+/db mice (Supplementary Figure 4B). Interestingly, only splenocytes isolated from db/db CD362^−^ and db/db CD362^+^ mice led to a lower collagen production upon co-culture with fibroblasts vs. splenocytes from db/db animals.

## Discussion

The salient findings of the present study are that all the MSC populations tested in this experimental model of type 2 diabetes mellitus-associated diabetic cardiomyopathy, characterized by diastolic dysfunction and absence of cardiac fibrosis, are able to reduce cardiomyocyte stiffness and restore the impaired underlying NO-cGMP signaling cascade. Interestingly, these effects tended to be less pronounced after CD362^+^ MSC application. Additionally, increased arteriole density was more pronounced after WT and CD362^−^ MSCs compared with CD362^+^ MSC application to db/db mice; however, all MSCs exerted systemic immunomodulatory effects and resulted in reduced immune cell presence in the heart. These observations together with our previous findings showing an improvement in diastolic dysfunction only after WT and CD362^−^ MSC application in db/db mice (25) indicate that in a model of diastolic dysfunction in the absence of cardiac fibrosis, a specific degree of decrease in cardiomyocyte stiffness and restoration of the underlying NO-cGMP-PKG-titin phosphorylation pathway is required to ameliorate diastolic dysfunction.

Evidence from a rodent model of the metabolic syndrome and from type I diabetic mice (11) indicates that dysregulation of the sarcomere protein titin is sufficient to induce diastolic dysfunction, even in the absence of cardiac fibrosis (9, 35). We previously showed lower PKG levels and titin hypophosphorylation in db/db mice, which is reflected in diastolic dysfunction (25). Assessment of NO and cGMP levels and cardiomyocyte stiffness in the present study corroborates the hypothesis that impaired NO-cGMP-PKG signaling leads to titin hypophosphorylation and cardiomyocyte stiffness (12, 14), as indicated by the high F_passive_ in isolated cardiomyocytes of db/db mice. Unaltered collagen I and III protein expression compared with non-diabetic db/db+ mice further illustrates that the diastolic dysfunction in this model of early diabetic cardiomyopathy occurs in the absence of cardiac fibrosis. With respect to stromal cell application, we recently demonstrated that only application of WT and CD362^−^ MSCs normalized PKG activity and titin phosphorylation in db/db mice and was associated with restored diastolic performance in these animals (25). Based on our previous findings illustrating that impaired NO bioavailability, a hallmark of endothelial dysfunction (13, 36, 37), could be increased by i.v. application of placenta-derived stromal cells (11), we suggested that the improved diastolic performance following WT and CD362^−^ MSC administration (25) was caused by changes of the NO-cGMP-PKG pathway. Supporting this hypothesis, we observed normalized NO and cGMP levels in db/db mice upon cell application; however, this effect was less pronounced in CD362^+^ MSC-treated db/db mice. Beyond NO-dependent PKG activation, vascularization can also be increased by MSCs (11). Accordingly, increased arteriole density was detected in db/db mice injected with WT, CD362^−^, and CD362^+^ MSCs. In parallel to the smaller increase in NO and cGMP levels, db/db mice that received CD362^+^ MSCs displayed a lower number of arterioles than did the db/db CD362^−^ group. This finding is supported by the study of De Rossi et al. (38), who demonstrated that released syndecan-2 (=CD362) induces anti-angiogenic effects. It further suggests that less pronounced pro-angiogenic/endothelial-protective effects of CD362^+^ MSCs compared with WT and CD362^−^ MSCs underlie the lower PKG activity, subsequent titin hypophosphorylation, lower improvement in cardiomyocyte stiffness, and failure to improve diastolic function in db/db mice.

Given the well-accepted concept of crosstalk between inflammation and cardiac fibrosis on the one hand (39) and the immunomodulatory properties of MSCs on the other hand (17, 22), we investigated the impact of MSC application on cardiac and splenic immune cell presence. In agreement with their ability to decrease the number of immune cells in the heart (17), WT as well as CD362^−^ and CD362^+^ MSCs reduced cardiac CD3^+^ and CD68^+^ cell presence in db/db mice. Furthermore, application of all MSC populations led to fewer apoptotic Tregs within the spleen; however, only CD362^+^ MSCs increased the percentage of splenic Tregs in db/db mice. In line with observations in ob/ob mice (40), db/db animals displayed higher numbers of splenic pro-inflammatory CD4^+^IFN-γ^+^ and CD8^+^IFN-γ^+^ cells, whereas the numbers of anti-inflammatory CD4^+^IL-10^+^ and CD8^+^IL-10^+^ cells were reduced. MSC application modulated the percentages of these specific immune cells in the spleen, with the observed effects differing depending on the applied MSC population. The immunomodulatory effects observed following WT and CD362^+^ MSC application were similar; both cell populations increased the anti-inflammatory splenic CD4^+^IL-10^+^ and CD8^+^IL-10^+^ cells in db/db mice, whereas CD362^−^ MSCs led to a decrease. Injection with CD362^−^ MSCs also led to the largest reduction of pro-inflammatory cells in the MSC-treated db/db animals. Since syndecans are known to interact with immune cells (41), the absence of CD362 may explain the differences in those immunomodulatory effects following CD362^−^ vs. WT and CD362^+^ MSC application. However, further investigations beyond the scope of this study are needed to support this hypothesis. In general, a reduction in systemic and cardiac inflammatory cells was observed after MSC application, which might in part be explained by the MSC-mediated increase in Tregs and/or Tregs quality (reduction in apoptotic Tregs) (42). However, a direct MSC/CD362-mediated effect on the specific analyzed T cells cannot be excluded.

Given the relevance of the cardiosplenic axis in the pathogenesis of heart failure, we investigated the pro-fibrotic potential of splenocytes derived from the different groups. Similar to splenocytes isolated from streptozotocin-induced diabetic cardiomyopathy mice (11), db/db-derived splenocytes increased the collagen production of fibroblasts upon co-culture. Interestingly, splenocytes from CD362^−^ and CD362^+^ db/db mice exhibited a lower pro-fibrotic potential than did splenocytes of db/db mice. Given the early-onset model of experimental diabetic cardiomyopathy without cardiac fibrosis, we hypothesize that the CD362^−^- and CD362^+^-mediated immunomodulatory and anti-fibrotic effects may be reflected in a reduction in cardiac fibrosis at a later stage.

Importantly, all the abovementioned effects following the different applied stromal cells in db/db mice occurred in the absence of changes in BG or HbA_1c_ levels. This is in contrast to previous studies in humans (43–45) and rodents (19, 20, 46) in which a therapeutic effect on glycemic control after stromal cell treatment was found. This phenomenon was explained by the regenerative properties of MSCs, including their capacity to differentiate into insulin-producing cells (19) and their ability to repair pancreatic β-cells in a paracrine manner (47, 48). Most of these studies were performed with murine MSCs or with human MSCs in immunodeficient [severe combined immunodeficiency (SCID)] mice. We suggest that absence of alterations in BG or HbA_1c_ levels after MSC application in db/db mice might be explained by the xenogenic approach in non-immunocompromised mice, leading to a lower regenerative capacity.

## Conclusion

The i.v. application of CD362^+^ MSCs resulted in reduced cardiomyocyte stiffness paralleled by elevated NO and cGMP levels and increased arteriole density in a model of early-onset diabetic cardiomyopathy without fibrosis. These effects were less pronounced than after WT and CD362^−^ MSC administration, known to improve diastolic performance in db/db mice (25). These findings indicate that the CD362^+^ MSC-mediated decrease in cardiomyocyte stiffness and increase in NO-cGMP levels were insufficient to improve diastolic function in this model.

## Data Availability Statement

The original contributions presented in the study are included in the article/Supplementary Material, further inquiries can be directed to the corresponding author/s.

## Ethics Statement

The animal study was reviewed and approved by the Landesamt für Gesundheit und Soziales, Berlin, Germany (G0254/13).

## Author Contributions

KP wrote the manuscript and substantially contributed to data acquisition including data analysis and interpretation, and intellectual content. FD, KM, AK, ME-S, and BK contributed to data acquisition and data analysis. LO'F and SE provided the human MSCs used in the study, including data of cell characterization. NH, SE, and TO'B revised the manuscript. CT coordinated funding and revised the manuscript. SVL performed conception of the study, data analysis and interpretation, coordinated funding, and revised the manuscript. All authors revised the manuscript for intellectual content and gave their final approval for publication.

## Conflict of Interest

SE is the Chief Scientific Officer and LO'F is the Head of Process Development at Orbsen Therapeutics Ltd. (Galway, Ireland), a company that is developing the CD362+ MSC for therapeutic purposes. The remaining authors declare that the research was conducted in the absence of any commercial or financial relationships that could be construed as a potential conflict of interest.
