# Supplementary material for: Impact of Syndecan-2-Selected Mesenchymal Stromal Cells on the Early Onset of Diabetic Cardiomyopathy in Diabetic db/db Mice
Source: Front Cardiovasc Med. 2021 May 21;8:632728. doi: 10.3389/fcvm.2021.632728 (PMC8175674; doi:10.3389/fcvm.2021.632728)
Supplement: Supplementary file 1 [file Data_Sheet_1.docx]

Supplemental material

Impact of syndecan-2-selected mesenchymal stromal cells on the early onset of diabetic cardiomyopathy in diabetic db/db mice

Kathleen Pappritz^1,2,3^, Fengquan Dong^2^, Kapka Miteva^2,4^, Arpad Kovacs^5^, Muhammad El-Shafeey^1,2,3,6^, Bahtiyar Kerim^2^, Lisa O’Flynn^7^, Steve Elliman^7^, Timothy O’Brien^8^, Nazha Hamdani^5,9,10^, Carsten Tschöpe^1,2,3,11^, Sophie Van Linthout^1,2,3*^

^1^Berlin Institute of Health at Charité - Universitätmedizin Berlin; BIH Center for Regenerative Therapies (BCRT), Berlin, Germany

^2^ Berlin-Brandenburg Center for Regenerative Therapies, Charité, Universitätsmedizin Berlin, Berlin, Germany

^3^German Center for Cardiovascular Research (DZHK), Partner site Berlin, Berlin, Germany

^4^Division of Cardiology, Foundation for Medical Research, Department of Medicine Specialized Medicine, Faculty of Medicine, University of Geneva, Geneva, Switzerland

^5^ Institute of Physiology, Ruhr University Bochum, Bochum, Germany

^6^Medical Biotechnology Research Department, Genetic Engineering and Biotechnology Research Institute (GEBRI), City of Scientific Research and Technological Applications, Alexandria, Egypt

^7^Orbsen therapeutics, National University of Ireland Galway, Galway, Ireland

^8^Regenerative Medicine Institute and Department of Medicine, National University of Ireland Galway, Galway, Ireland

^9^Molecular and Experimental Cardiology, Ruhr University Bochum, Bochum, Germany

^10^Department of Cardiology, St. Josef-Hospital, Ruhr University Bochum, Bochum, Germany

^11^Department of Cardiology, Charité - Universitätsmedizin Berlin, Campus Virchow Klinikum, Berlin, Germany

*** Correspondence:**Sophie Van Linthout, PhD

Berlin Institute of Health at Charité - Universitätmedizin Berlin

BIH Center for Regenerative Therapies (BCRT)

Charitéplatz 1

10117 Berlin, Germany

Phone: +49-(0)30-450539486

Fax: +49-(0)30-450539409

sophie.van-linthout@charite.de

Supplemental Figure 1


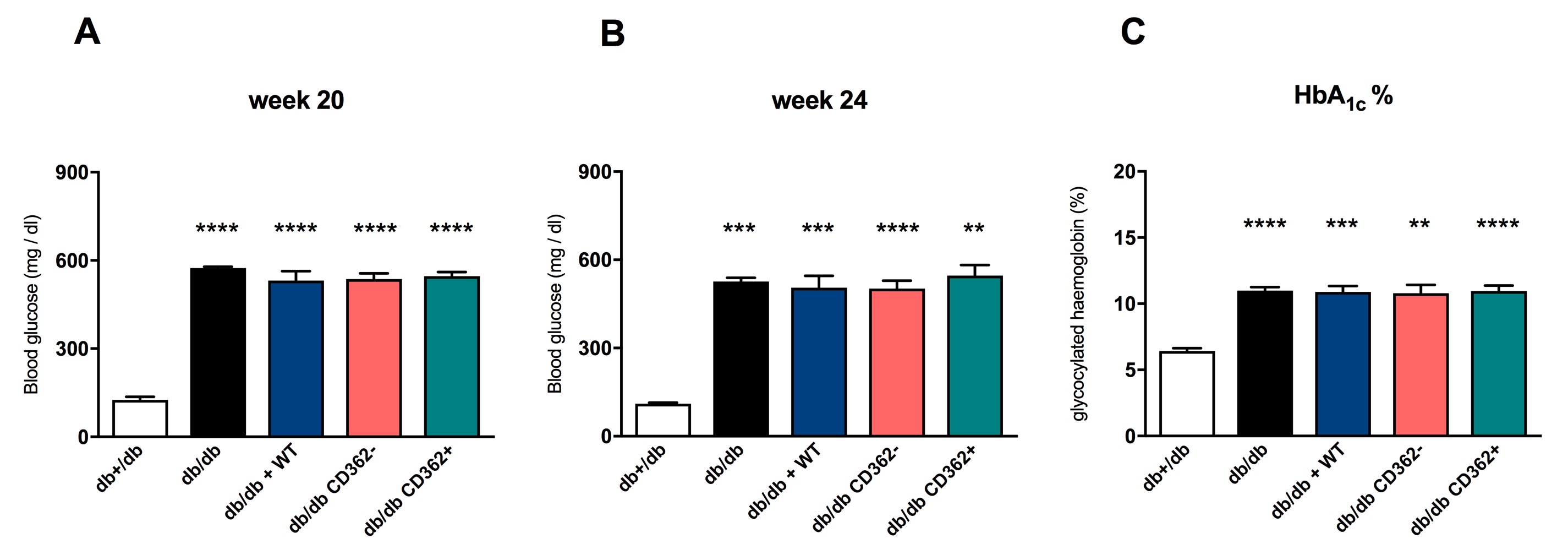


**Supplemental Figure 1. Application of WT, CD362^-^, and CD362^+^ MSC do not affect blood glucose and HbA1c levels of db/db mice.** Bar graphs represent the mean±SEM of blood glucose levels (mg/dl) before cell application, at an age of 20 weeks (**A**), 4 weeks after cell application, at an age of 24 weeks (**B**) and glycated hemoglobin (HbA_1c_), 4 weeks after cell application (**C**) depicted as percentage (%) in db+/db, db/db, and db/db mice receiving WT, CD362^-^ or CD362^+^ MSC. Data were analyzed with One-way ANOVA or Welch-ANOVA (^**^p<0.01, ^***^p<0.001, ^****^p<0.0001 versus db+/db; BG: n=10 for db+/db, n=9 for db/db but only n=3 were detectable, n=10 for db/db WT but only n=6 were detectable, n=10 for db/db CD362^-^ but only n=7 were detectable, and n=11 for db/db CD362^+^ group but only n=4 were detectable; HbA_1c_: n=5-6/group). The undetectable glucose levels were above the detection limit.

Supplemental Figure 2


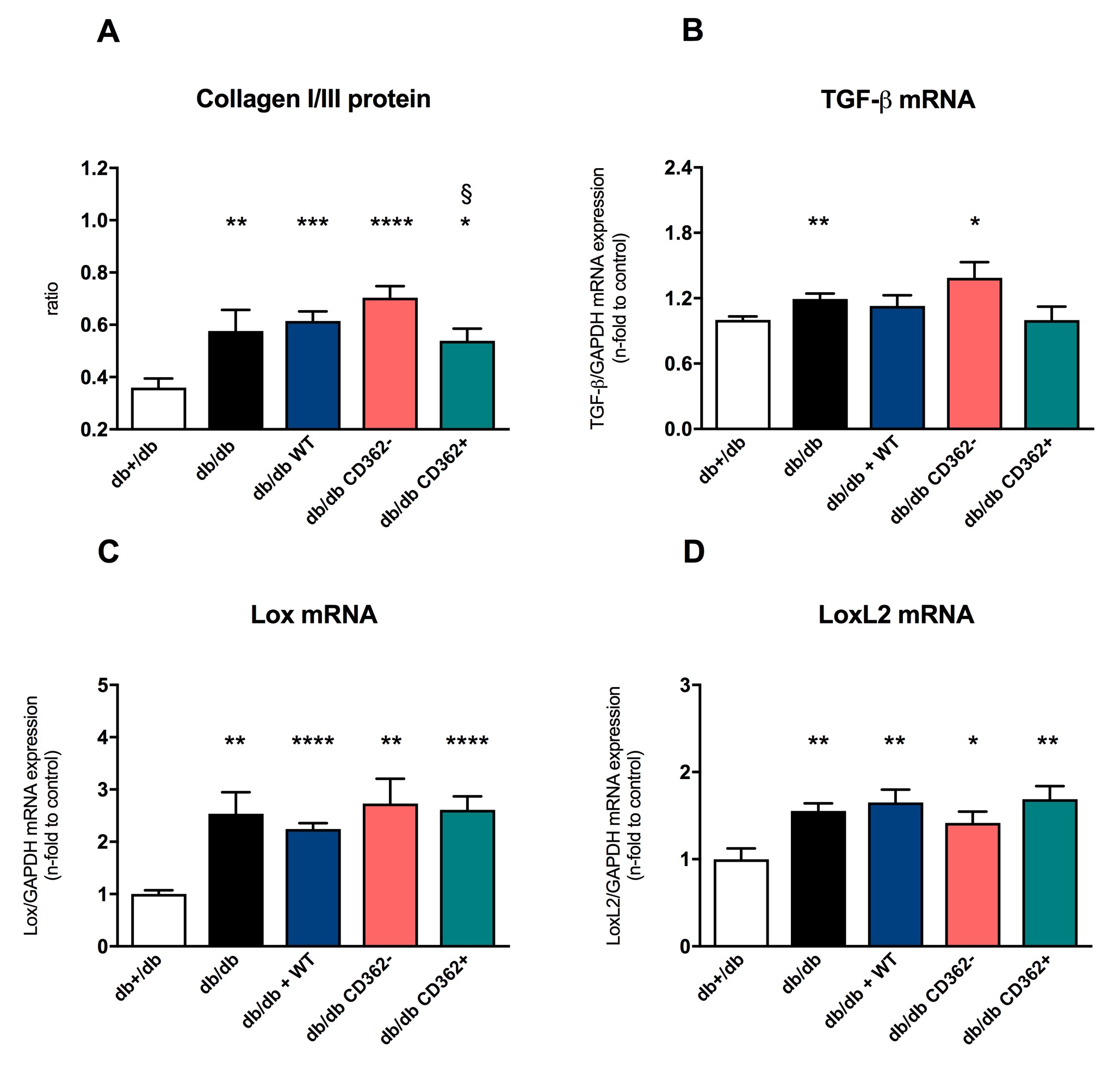


**Supplemental Figure 2. Application of WT, CD362^-^, and CD362^+^ MSC do not influence the cardiac collagen ratio and gene expression of fibrosis-associated genes in db/db mice.** (**A**) After quantification of cardiac collagen I and III protein expression, the collagen I/III ratio was calculated. To further characterize myocardial fibrosis, gene expression of pro-fibrotic transforming growth factor (TGF)-β (**B**) and the collagen cross-linking enzymes lysyl oxidase (Lox, **C**), and Lox-like (LoxL)-2 (**D**) were measured. Data were depicted as mean±SEM and analyzed with One-way ANOVA or Welch-ANOVA (^*^p<0.05, ^**^p<0.01, ^***^p<0.001, ^****^p<0.0001 versus db+/db; ^§^p<0.05 versus db/db CD362- with n=11 for db+/db, n=8-9 for db/db, n=9-10 for db/db WT, n=9-10 for db/db CD362^-^, and n=10-11 for db/db CD362^+^).

Supplemental Figure 3


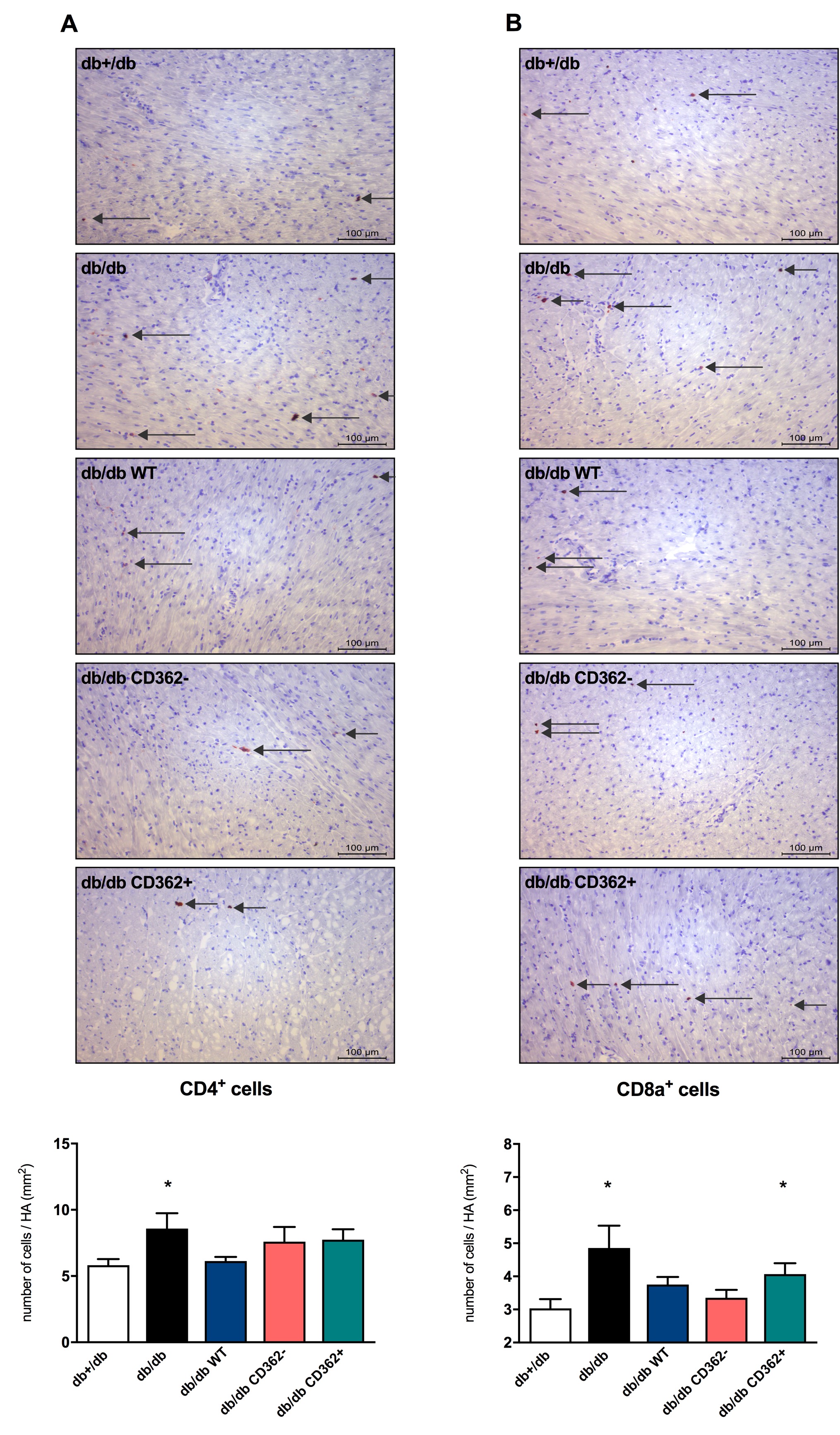


**Supplemental Figure 3. Application of WT, CD362^-^, and CD362^+^ MSC does not affect myocardial presence of CD4^+^ and CD8^+^ immune cells in db/db mice.** Characterization of the immune cells present within the myocardium 4 weeks after stromal cell administration. Upper panel: representative immunohistological staining (scale bar = 100μm) of CD4^+^ (**A**) and CD8a^+^ (**B**) cells. In the graphs below, the respective quantification performed via digital image analysis is depicted. Bar graphs represent the mean±SEM of the number of cells/heart area (HA, mm^2^) and were analyzed with One-way ANOVA or Welch-ANOVA (^*^p<0.05 versus db+/db; with n=11 for db+/db, n=7 for db/db, n=8 for db/db WT, n=8 for db/db CD362^-^, and n=11 for db/db CD362^+^ group).

Supplemental Figure 4


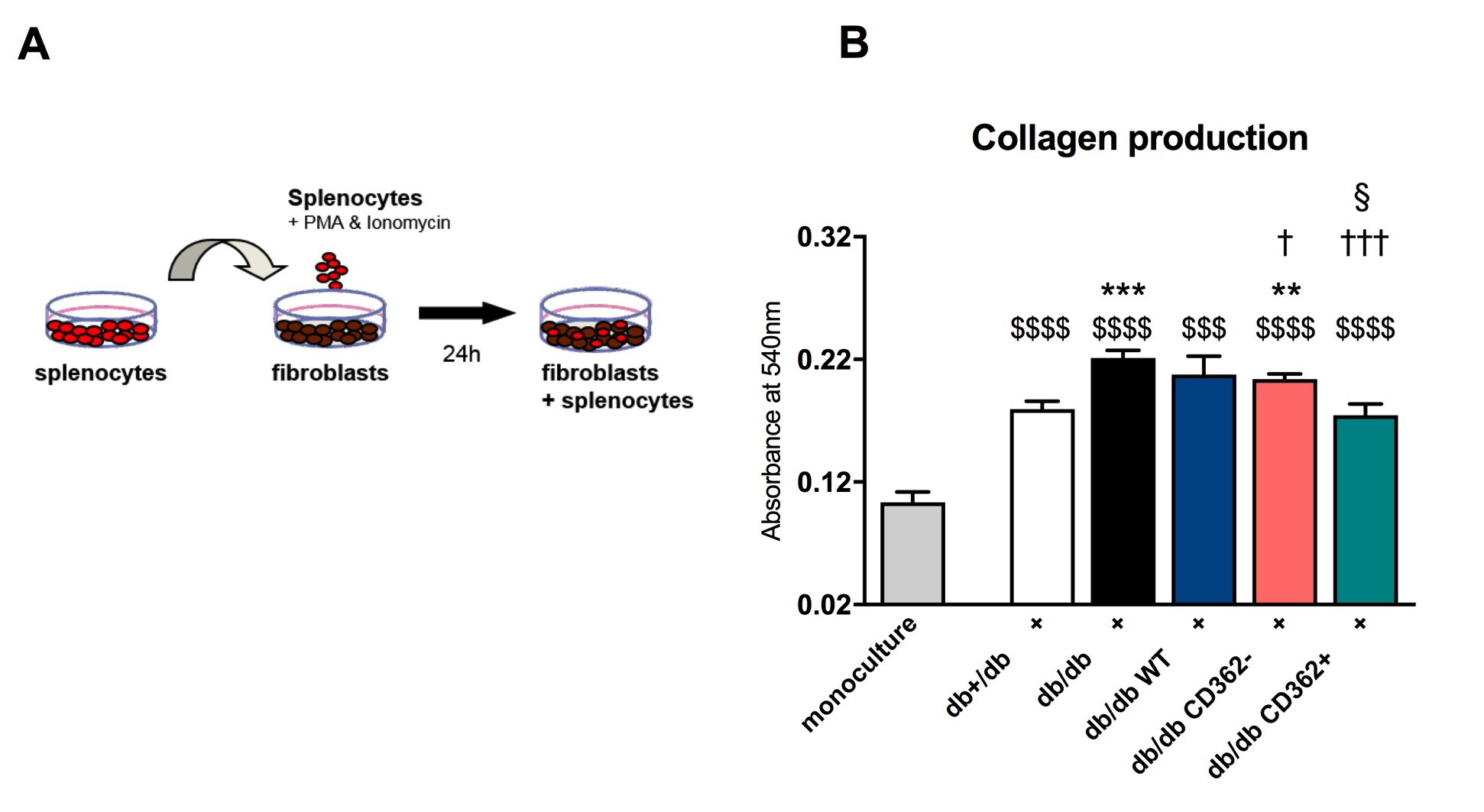


**Supplemental Figure 4. Application of CD362^-^ and CD362^+^ MSC reduce the pro-fibrotic potential of splenocytes derived from db/db mice.** (**A**) Experimental set-up to assess the pro-fibrotic potential of splenocytes co-cultured with fibroblasts. To this end, collagen production in fibroblasts was measured using Sirius red staining after 24 h of co-culture. (**B**) Quantification of the photometric analyses with n=11 for the monoculture, n=10 for splenocytes derived from db+/db, n=9 for splenocytes derived from db/db, n=6 for splenocytes derived from db/db WT, n=8 for splenocytes derived from db/db CD362^-^, and n=9 for splenocytes derived from db/db CD362^+^ group. Data were depicted as mean±SEM and analyzed with Welch-ANOVA (^$$$^p<0.001, ^$$$$^p<0.0001 versus monoculture; ^**^p<0.01, ^***^p<0.001 versus db+/db; ^†^p<0.05, ^†††^p<0.001 versus db/db; ^§^p<0.05 versus db/db CD362^-^).
